# Parallel comparison of R.E.N.A.L., PADUA, and C‐index scoring systems in predicting outcomes after partial nephrectomy: A systematic review and meta‐analysis

**DOI:** 10.1002/cam4.4047

**Published:** 2021-07-14

**Authors:** Can Hu, Jiale Sun, Zhiyu Zhang, Haoyang Zhang, Qi Zhou, Jiangnan Xu, Zhixin Ling, Jun Ouyang

**Affiliations:** ^1^ Department of Urology The First Affiliated Hospital of Soochow University Suzhou China

**Keywords:** C‐index, clinical outcomes, PADUA, partial nephrectomy, R.E.N.A.L.

## Abstract

**Objective:**

To parallelly compare the applicability of the radius, exophytic/endophytic, nearness, anterior/posterior, location nephrometry score (R.E.N.A.L.), the Preoperative Aspects and Dimensions Used for an Anatomical (PADUA), and the centrality index (C‐index) scoring systems in predicting clinical outcomes after partial nephrectomy (PN).

**Methods:**

We searched EMBASE, PubMed, Ovid, and Web of Science to perform a meta‐analysis examining the correlation coefficients between three nephrometry scores (NSs) and warm ischemia time (WIT), estimated blood loss (EBL), operation time (OT), length of stay (LOS), and absolute change in eGFR (ACE) up to 25 January 2021.

**Results:**

In total, 13 studies including 1496 patients met the criteria for further analysis. Overall, all scoring systems had statistically significant correlations with the WIT, EBL, OT, ACE and LOS and ACE, except for the correlation between PADUA and LOS (*r* = 0.16 [−0.00, 0.31], *p *> 0.05). The C‐index had the strongest correlation with WIT (*r* = −0.35 [−0.43, −0.26], *p *< 0.05) and ACE (*r* = −0.29 [−0.48, −0.10], *p *< 0.05). Weak correlations were observed between OT as well as EBL and each scoring system. Publication bias was observed in PADUA score predicting ACE (*p *= 0.04) and high heterogeneity was found in some of our results.

**Conclusion:**

Until now, this is the first meta‐analysis that parallelly compares these three scoring systems in predicting outcomes after PN. We found that all NSs showed a statistically significant correlation with WIT, EBL, OT, and ACE. Moreover, the C‐index scoring system is the best predictor of WIT and ACE. Due to the existence of publication bias and high heterogeneity, more well‐designed and large‐scale studies are warranted for validation.

## Abstract 2

To our knowledge, this is the first meta‐analysis that parallelly compares these three scoring systems in predicting outcomes after PN. Overall, three scoring systems were significantly correlated with WIT, EBL, OT and ACE. Moreover, the C‐index scoring system outperformed R.E.N.A.L. and PADUA scoring systems in WIT and ACE.

## 1 INTRODUCTION

Up to now, the morbidity of renal cell carcinoma (RCC) ranked sixth in men and ranked eighth in women among all tumors, respectively.^1^ Nevertheless, in clinical practice, the vast majority of patients with RCC remain symptom‐free even in their late period. Thanks to the widespread application of radiological imaging (e.g., ultrasonography and computerized tomography), the cases of RCC could be more frequently detected.^2^ Although there are various types of therapies for RCC of different stages, surgery is the only effective treatment for localized RCC.^3^ Thus, radical nephrectomy (RN) and partial nephrectomy (PN) were established successively. For clinically localized RCC, previous studies have demonstrated that PN was superior to RN due to its advantages of preservation of general kidney function and lower risk of metabolic and cardiovascular complications.^4^, ^5^, ^6^ However, in comparison with RN, PN is a more challenging procedure for surgeons, especially in complex renal tumor. Therefore, evaluating its complexity before PN is of great necessity.

In the past 10 years, several nephrometry scores (NSs) have been put forward to standardize renal masses and assist in the decision of surgical strategies. Among them, the radius, exophytic/endophytic, nearness, anterior/posterior, location nephrometry score (R.E.N.A.L.),^7^ the Preoperative Aspects and Dimensions Used for an Anatomical (PADUA),^8^ and the centrality index (C‐index)^9^ are the most known and widely used systems in the world. The R.E.N.A.L. and PADUA scoring systems based on semiquantitative anatomical factors and methodologies were both proposed in 2009. The C‐index score which was first mentioned in 2010 reflects the geometric correlation between the tumor and kidney. In conclusion, all these scoring systems based on radiological imaging are aimed to assist surgeons to determine the surgical strategies and facilitate outcome assessment.^10^ Although a quantity of studies has been published to validate the correlation between these NSs and clinical outcomes, it still remains controversial which scoring system could most accurately assess the outcomes of PN. Therefore, we conducted a systematic evaluation and meta‐analysis to parallelly compare the role of three scoring systems in predicting the perioperative and postoperative results of PN.

## 2 MATERIALS AND METHODS

### 2.1 Search strategies

A comprehensive search was conducted via PubMed, Ovid, EMBASE, and Web of Science databases for the following key words: ([nephrometry] OR [The radius, exophytic/endophytic, nearness, anterior/posterior, location score] OR [R.E.N.A.L.] AND [the Preoperative Aspects and Dimensions Used for an Anatomical score] OR [PADUA] AND [centrality index] OR [C‐index]) AND ([PN] OR [nephron sparing surgery]). The detailed syntax is shown in Supporting Information [Link], [Link]. The last search was up to 21 December 2020. Our meta‐analysis was reported according to the preferred reporting items of the system review and meta‐analysis (PRISMA).^11^

### 2.2 Inclusion and exclusion criteria

Inclusion criteria: (a) comparative studies which were conducted prospectively or retrospectively; (b) patients who underwent PN; (c) patients who were evaluated according to renal score as R.E.N.A.L., PADUA, and C‐index simultaneously; (d) perioperative and postoperative outcomes could be acquired; (e) correlation between NSs and outcomes was assessed by correlation coefficient.

Exclusion criteria: (a) data incomplete or invalid; (b) no interest outcome; (c) republished reports; (d) review articles and editorial comments; (e) case report or series.

### 2.3 Data extraction and study quality assessment

Two reviewers (Hu and Sun) independently screened the titles and abstracts of articles which met our inclusion criteria. Then, full‐text analysis was conducted to ensure inclusion. The controversy was resolved by negotiation or a senior reviewer. Extracted data included: study year, country, study design, number of patients, operation type, perioperative and postoperative outcomes. To avoid the risk of bias, two reviewers evaluated the quality of these nonrandomized studies independently by using Newcastle‐Ottawa Quality Assessment Scale (NOS).^12^ The total score ≤5 was considered low quality, six to seven intermediate quality and ≥8 high quality.

### 2.4 Outcomes assessed

At least one specific perioperative outcome after PN should be included in study, including, operation time (OT), estimated blood loss (EBL), length of stay (LOS), absolute change in estimated glomerular filtration rate (ACE), and warm ischemia time (WIT). Among them, WIT is defined as the duration of the renal artery being clamped^13^; eGFR is defined according to modification of diet in renal disease study group (MDRD)^14^ or radionuclide scans.

### 2.5 Statistical analysis

All statistical analyses and meta‐analyses were carried out using Stata (version 16; StataCorp, College Station, TX, USA). Subgroup analysis was performed according to three NSs. The effect size was assessed via correlation coefficient (*r*) and its 95% confidence interval (CI). For studies reporting Spearman correlation coefficient (*r*_s_) but not Pearson correlation coefficient (*r*_p_), the asymptotic relationship between *r*_s_ and *r*_p_ can be mathematically expressed by the formula: *r*_p_ = 2sin (*r*_s_π/6).^15^ Next, fisher's *r*‐to‐*z* transformation was conducted to calculate 95% CI of *r*_p_ (*r* stands for *r*_p_ in the following text). Heterogeneity was evaluated using inconsistency (*I* ^2^) statistics. *I* ^2^ > 50%, *I* ^2^ = 25%–50%, and *I* ^2^ < 50% was considered as high, moderate, low degree of variance, respectively.^16^ To evaluate the pooled estimate conservatively in this study, we used the random‐effects model regardless of *I* ^2^. Subsequently, to confirm the source of significant heterogeneity, sensitivity analysis was performed. In addition, publication bias was assessed by egger's test. All significance levels were set at two‐sides *p *< 0.05.

## 3 RESULTS

### 3.1 Characteristics of included studies and quality assessment

**FIGURE 1:**
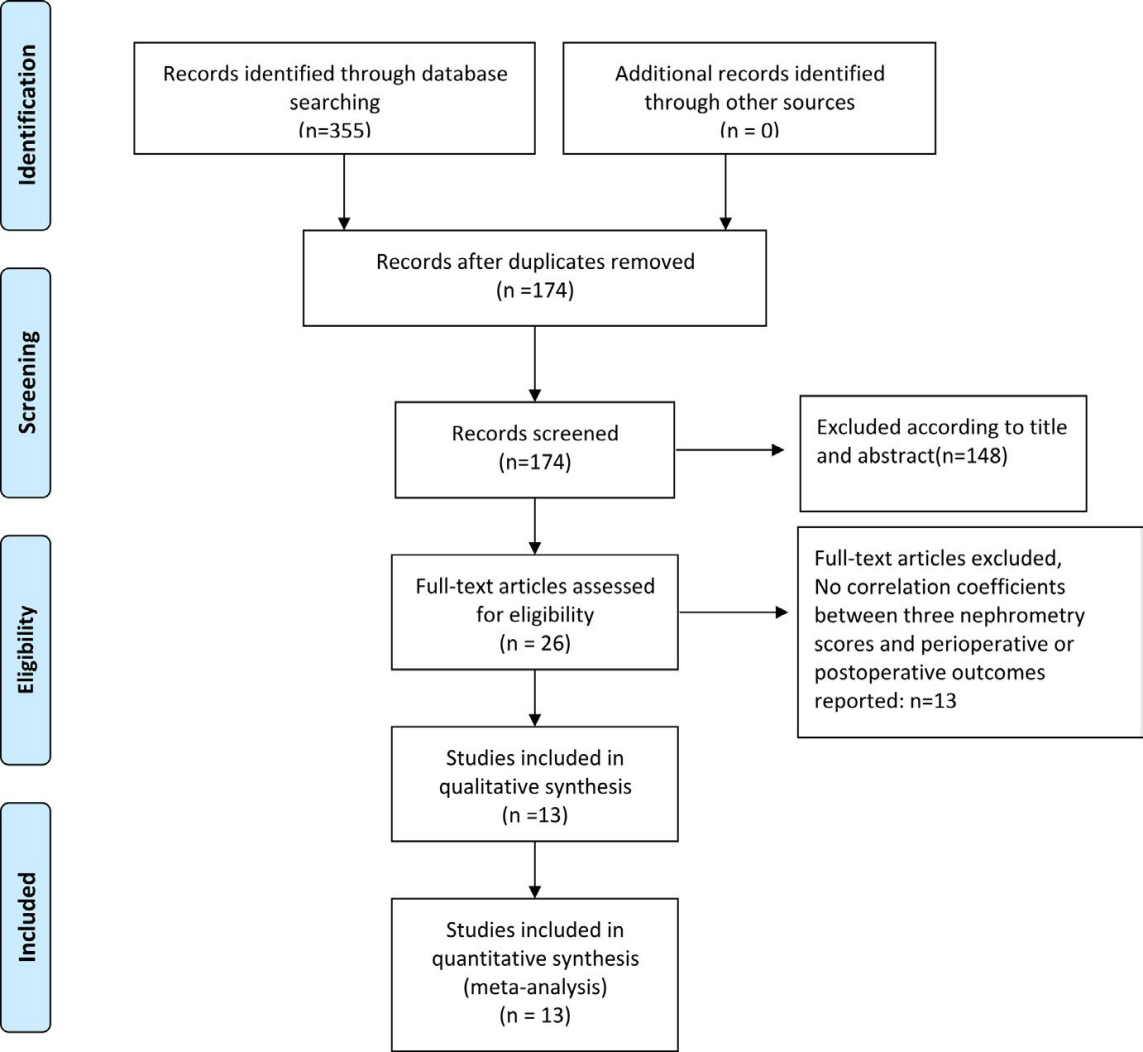
PRISMA 2009 Flow Diagram

The screening process was summarized visually by the PRISMA flow diagram (Figure 1). At last, with a total of 1496 patients, 13 studies^17^, ^18^, ^19^, ^20^, ^21^, ^22^, ^23^, ^24^, ^25^, ^26^, ^27^, ^28^, ^29^, ^30^, ^31^, ^32^ published between 2006 and 2018 were included in the meta‐analysis (Table 1). Among them, 12 were retrospective and 1 did not mention the research type. Besides, only four studies mentioned the surgical approach. Patients in three literature of these studies were performed with retroperitoneal^17^, ^20^, ^22^ approach and one had a transabdominal approach.^27^ In addition, the NOS scale demonstrated that the quality of our included studies varied from moderate to high (Supporting Information 1).

**TABLE 1:** Summary of the included studies

Authors	Study period	Country	Study design	Surgical approach	Sample size (*n*)
Kaan Karamik (2020)	2015–2018	Turkey	Retrospective study	LPN/RPN	78
Jingchao Liu (2020)	2016–2018	China	Retrospective study	LPN	135
Yu‐De wang (2020)	2013–2017	China	Retrospective study	OPN/LPN/RPN	40
Chan Ho Lee (2019)	NA	Korea	Retrospective study	OPN	162
Ergun Alma (2018)	2012–2017	Turkey	NA	OPN	132
Aditya P. Sharma (2017)	2014–2016	India	Retrospective study	LPN/RPN	50
Ravi M. Kumar (2017)	NA	Canada	Retrospective study	OPN/LPN	104
H. Borgmann (2016)	2009–2013	Germany	Retrospective study	OPN/RPN	188
Taekmin Kwon (2015)	2009–2011	Korea	Retrospective study	OPN/RPN	185
Massimiliano Spaliviero (2014)	2012–2014	US	Retrospective study	OPN/LPN/RPN	90
Linhui Wang (2014)	2012–2013	China	Retrospective study	RPN	69
Jason R. Bylund (2012)	2005–2011	US	Retrospective study	OPN/LPN/RPN	162
Zhamshid Okhunov (2011)	2006–2010	US	Retrospective study	LRP	101

Abbreviations: HLPN, hand‐assisted partial nephrectomy; LPN, laparoscopic partial nephrectomy; NA, not available; OPN, open partial nephrectomy; RPN, robotic‐assisted partial nephrectomy.

### 3.2 The correlations between three NSs and outcomes

#### 3.2.1 Warm ischemia time

**FIGURE 2:**
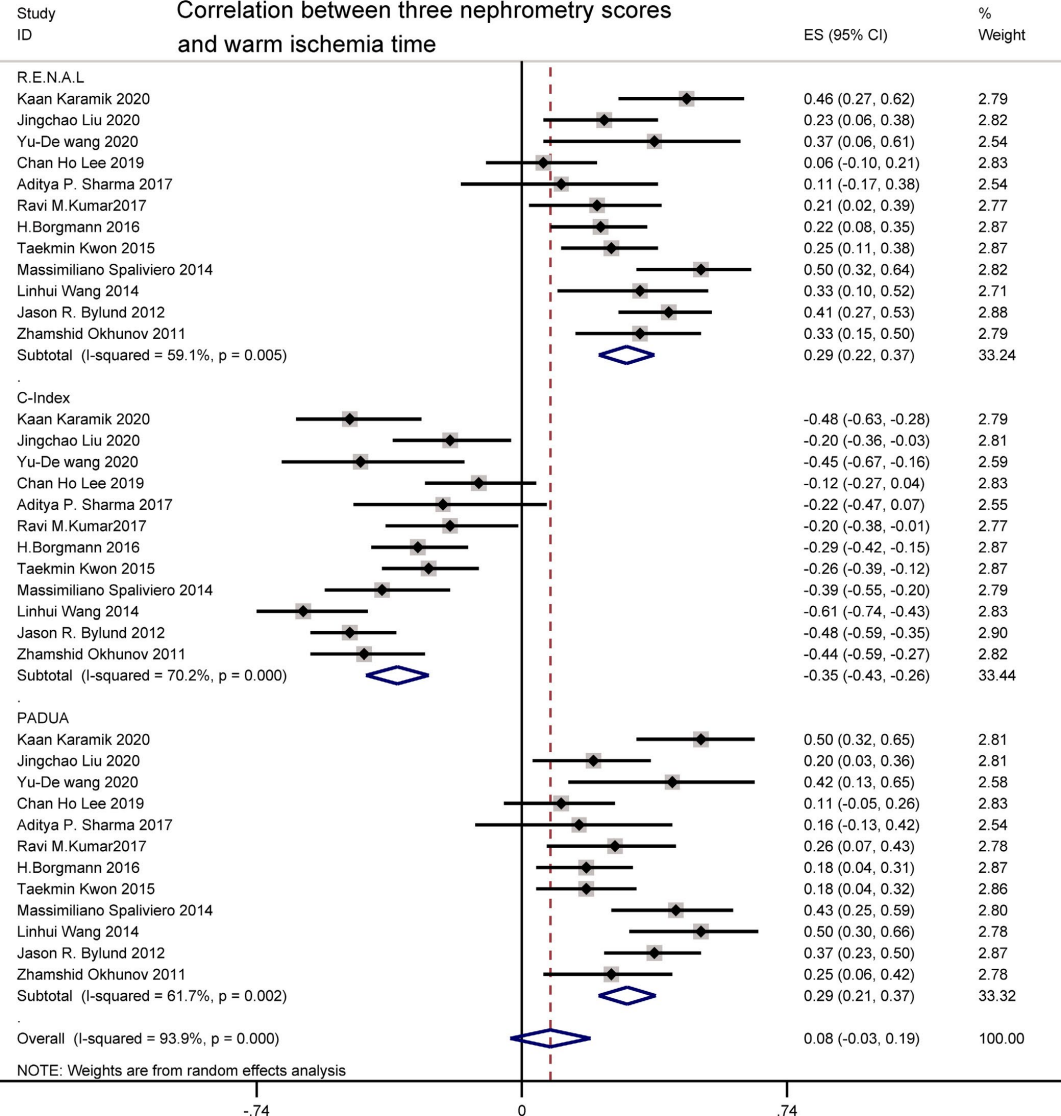
Correlation between three nephrometry scores and warm ischemia time

Overall, WIT was reported by 12 studies.^18^, ^19^, ^21^, ^22^, ^23^, ^24^, ^25^, ^28^, ^29^, ^30^, ^31^, ^32^ There was a weak significant association between WIT and R.E.N.A.L. and PADUA scoring systems (*r *= 0.29 [0.22, 0.37]; *I* ^2^ = 59.1%, *p *= 0.005; *r* = 0.29 [0.21, 0.37]; *I* ^2^ = 61.7%, *p *= 0.002). The C‐index score outperformed the other two, indicating a strong correlation with WIT (*r* = −0.35 [−0.43, −26]; *I* ^2^ = 70.2%, *p *< 0.001). For *I* ^2^ = 59.1%, 61.7% and 70.2%, high heterogeneity was found. Sources of heterogeneity needed to be carefully examined via sensitivity analysis (Figure 2).

#### 3.2.2 Estimated blood loss

**FIGURE 3:**
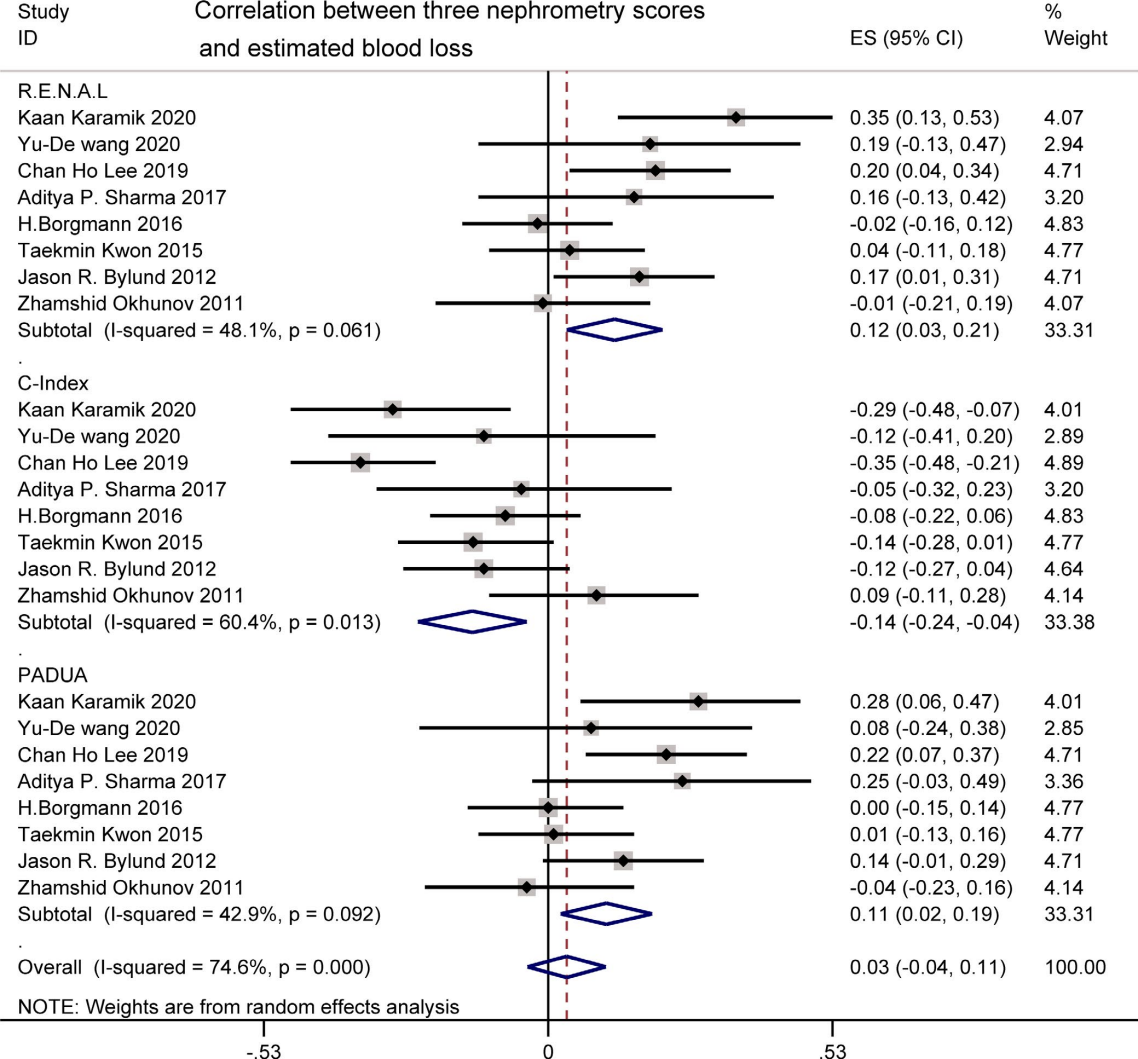
Correlation between three nephrometry scores and estimated blood loss

EBL was reported by eight studies.^18^, ^19^, ^21^, ^23^, ^24^, ^28^, ^29^, ^32^ A pooled analysis of R.E.N.A.L., PADUA, and C‐index scoring systems yielded a significant but weak relationship with EBL (*r *= 0.12 [0.03, 0.21]; *I* ^2^ = 48.1%, *p *= 0.061; *r* = 0.11 [0.02, 0.19]; *I* ^2^ = 42.9%, *p *= 0.092; *r* = −0.14 [−0.24, −0.04]; *I* ^2^ = 60.4%, *p *= 0.013). For *I* ^2^ = 60.4% in the C‐index, high heterogeneity was found. To confirm the source of heterogeneity, sensitivity analysis was performed (Figure 3).

#### 3.2.3 Operation time

**FIGURE 4:**
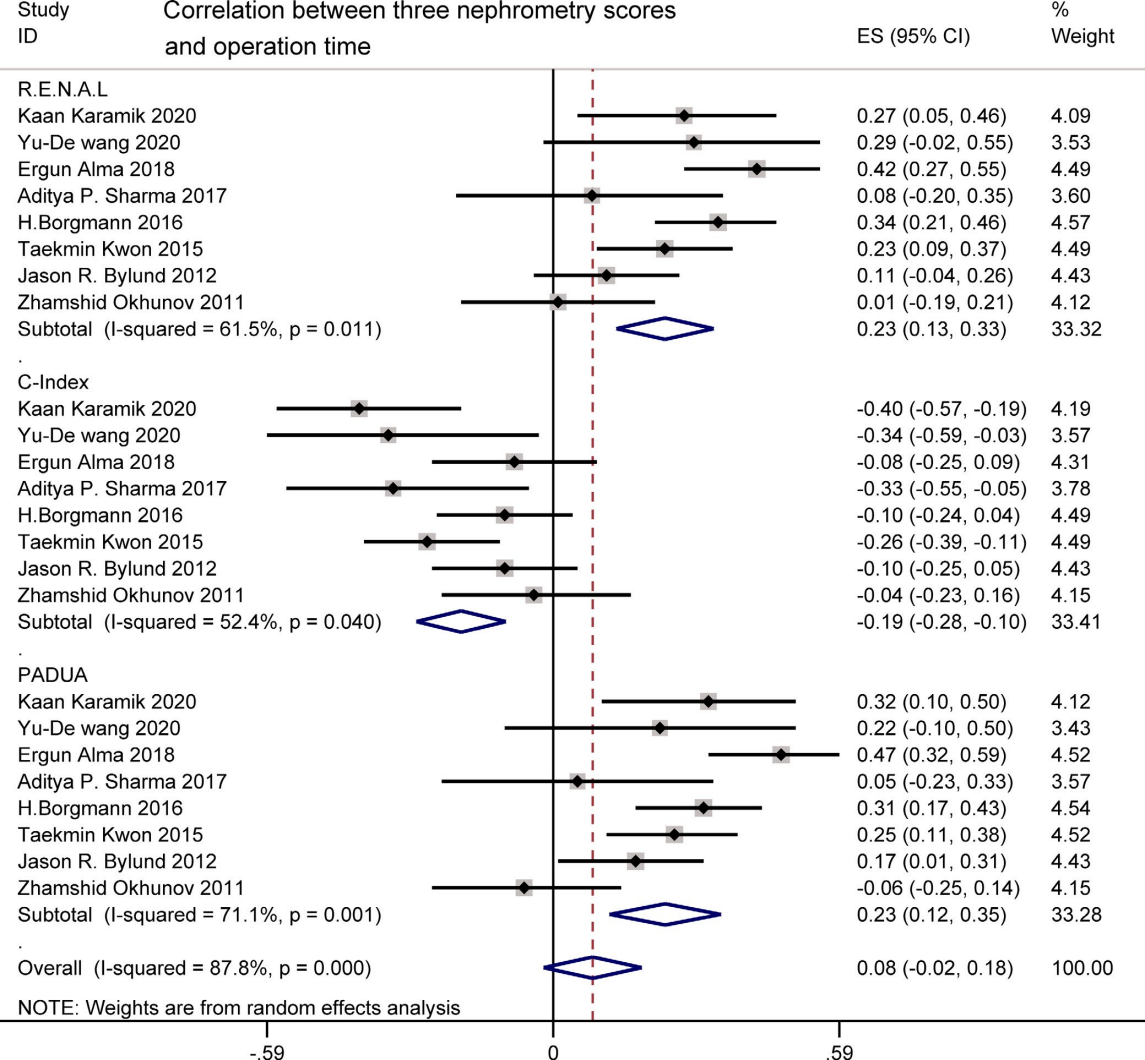
Correlation between three nephrometry scores and operation time

OT was reported by eight studies.^17^, ^18^, ^19^, ^21^, ^23^, ^28^, ^29^, ^32^ OT was correlated weakly with R.E.N.A.L. (*r* = 0.23 [0.13, 0.33]; *I* ^2^ = 61.5%, *p *= 0.011) and PADUA (*r *= 0.23 [0.12, 0.35]; *I* ^2^ = 71.1%, *p *= 0.001). There was also a weakly negative relationship between OT and C‐index score (*r* = −0.19 [−0.28, −0.10]; *I* ^2^ = 52.4%, *p *= 0.040). For *I* ^2^ = 61.5%, 87.8%, and 52.4%, high heterogeneity was found. Sources of heterogeneity required to be identified via sensitivity analysis (Figure 4).

#### 3.2.4 Absolute change in estimated glomerular filtration rate

**FIGURE 5:**
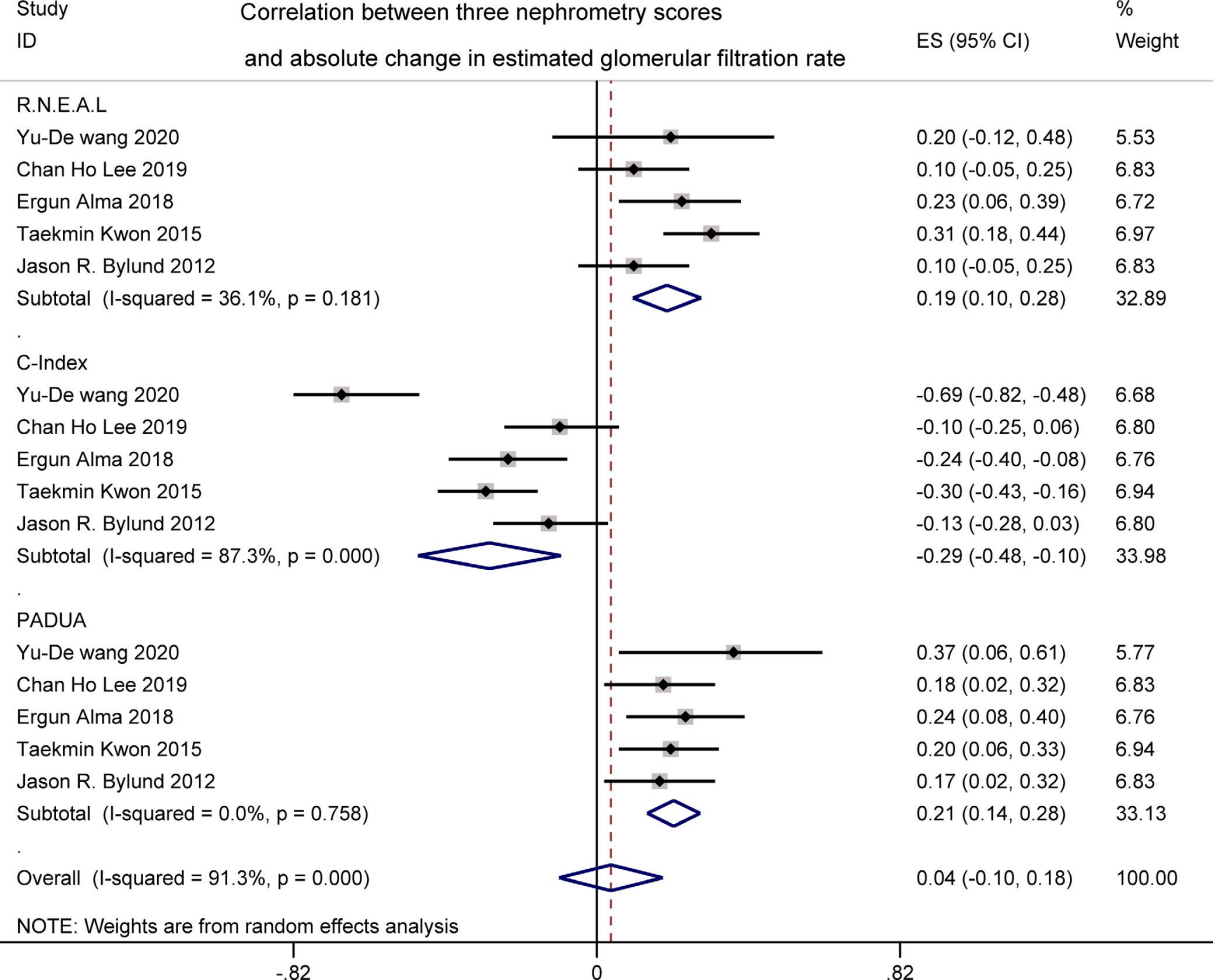
Correlation between three nephrometry scores and absolute change in estimated glomerular filtration rate

**FIGURE 6:**
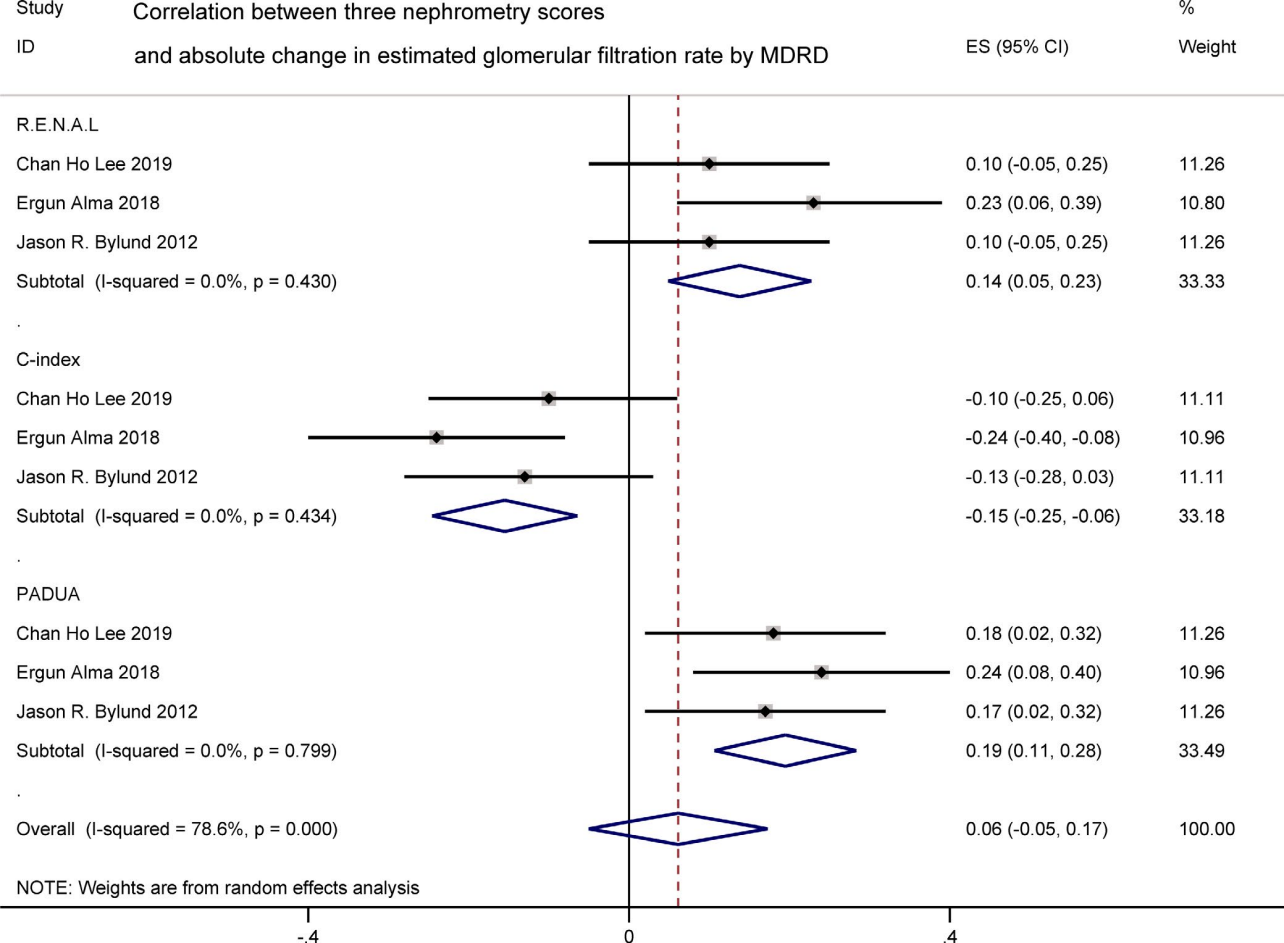
Correlation between three nephrometry scores and absolute change in estimated glomerular filtration rate evaluated by modification of diet in renal disease study group (MDRD)

**FIGURE 7:**
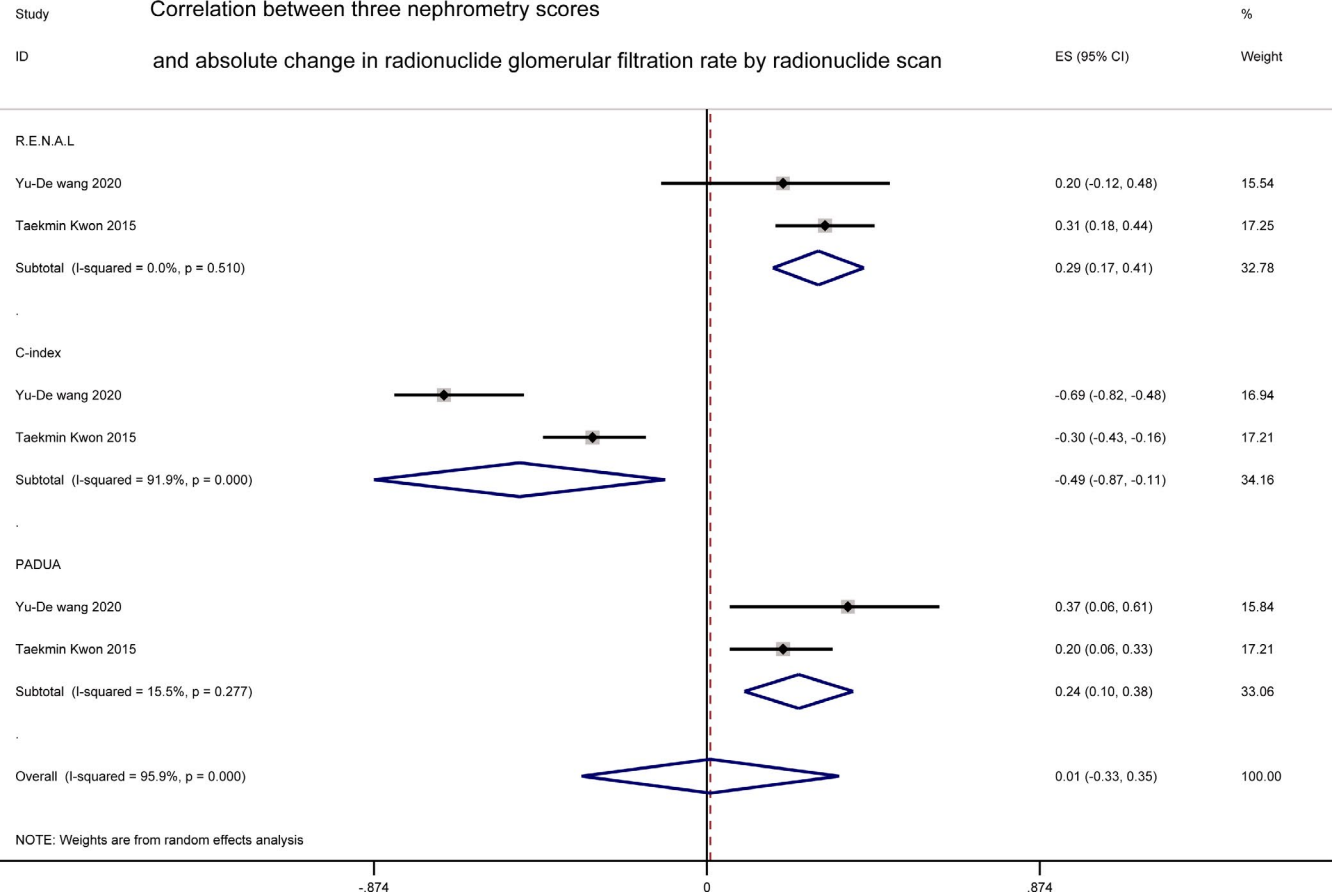
Correlation between three nephrometry scores and absolute change in estimated glomerular filtration rate by radionuclide scan

ACE was reported by five studies,^17^, ^19^, ^23^, ^24^, ^32^ showing a significant but weak relationship with R.E.N.A.L. (*r *= 0.19 [0.10, 0.28]; *I* ^2^ = 36.1%, *p *= 0.181) and PADUA (*r* = 0.21 [0.14, 0.28]; *I* ^2^ = 91.3%, *p *< 0.001). However, a pooled analysis of C‐index score yielded a relatively stronger relationship with ACE than the other two (*r* = −0.29 [−0.48, −0.10]; *I* ^2^ = 87.3%, *p *< 0.001). For *I* ^2^ = 91.3% and 87.3%, high heterogeneity was found. It was of great interest to explore the sources of heterogeneity among these studies (Figure 5). Due to the existence of heterogeneity, next we conducted the subgroup analysis by the two different methodologies of renal function evaluation. ACE evaluated by MDRD was reported by three studies,^17^, ^19^, ^24^ showing a weak correlation with R.E.N.A.L. (*r* = 0.14 [0.05, 0.23]; *I* ^2^ = 0.0%, *p *= 0.43), C‐index (*r *= −0.15 [−0.25, −0.06]; *I* ^2^ = 0.0%, *p *= 0.434), and PADUA (*r *= 0.19 [0.11, 0.28]; *I* ^2^ = 0.0%, *p *= 0.799) (Figure 6). ACE evaluated by radionuclide GFR was reported by two studies,^23^, ^32^ showing a significant relationship with R.E.N.A.L. (*r *= 0.29 [0.17, 0.44]; *I* ^2^ = 0.0%, *p *= 0.51) and PADUA (*r* = 0.24 [0.10, 0.38]; *I* ^2^ = 15.5%, *p *= 0.277). Moreover, a pooled analysis of C‐index score yielded a stronger relationship with ACE evaluated by radionuclide GFR than the other two (*r* = −0.49 [−0.87, −0.11]; *I* ^2^ = 91.9%, *p *< 0.001) (Figure 7).

#### 3.2.5 Length of stay

**FIGURE 8:**
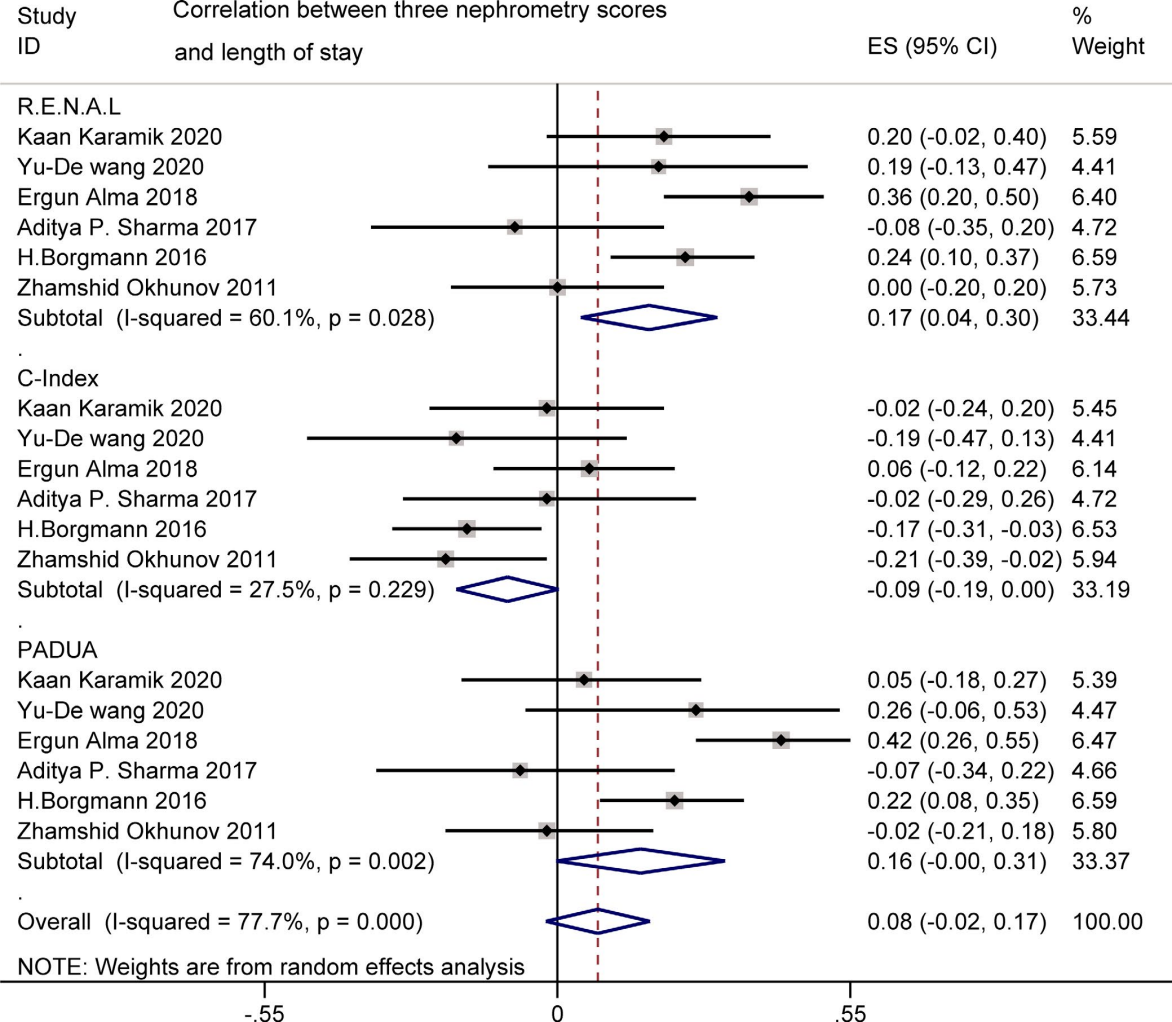
Correlation between three nephrometry scores and length of stay

LOS was reported by six studies.^17^, ^18^, ^21^, ^28^, ^29^, ^32^ Compared with the other two scores, A pooled analysis of PADUA score showed no statistically significant correlation with LOS (*r* = 0.16 [−0.00, 0.31], *p *> 0.05; *I* ^2 ^= 27.5%, *p *= 0.029), while R.E.N.A.L. (*r* = 0.17 [0.04, 0.30]; *I* ^2^ = 60.1%, *p *= 0.028) and C‐index (*r* = −0.09 [−0.19, −0.00]; *I* ^2 ^= 74.0%, *p *< 0.001) scores were found to have a weak positive correlation with LOS. For *I* ^2^ = 60.1% and 74.0%, high heterogeneity was found. Sources of heterogeneity needed to be identified via sensitivity analysis (Figure 8).

### 3.3 Sensitivity analysis

**FIGURE 9:**
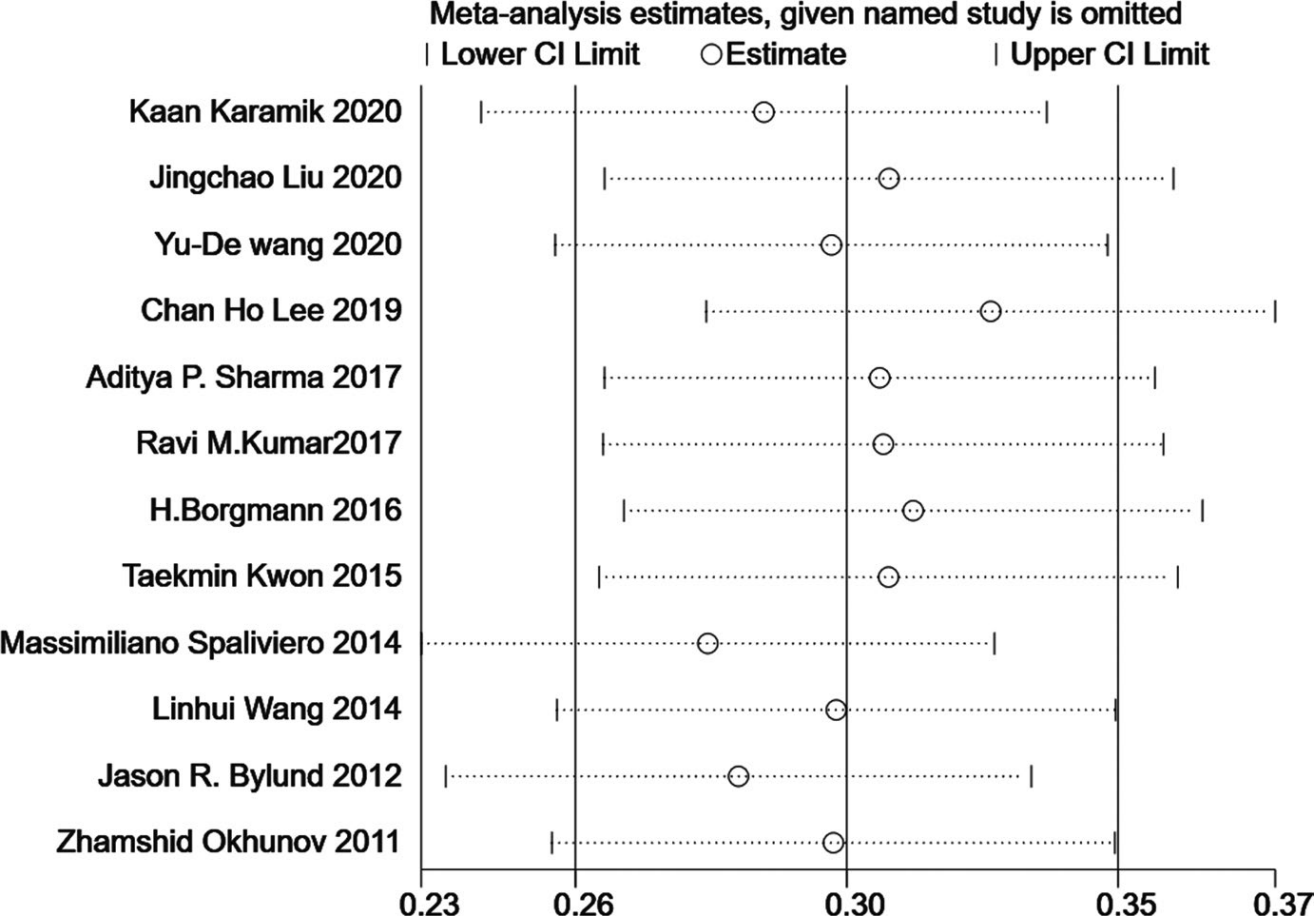
Sensitivity analysis of correlation between the radius, exophytic/endophytic, nearness, anterior/posterior, location nephrometry score (R.E.N.A.L.) scoring system and warm ischemia time

**FIGURE 10:**
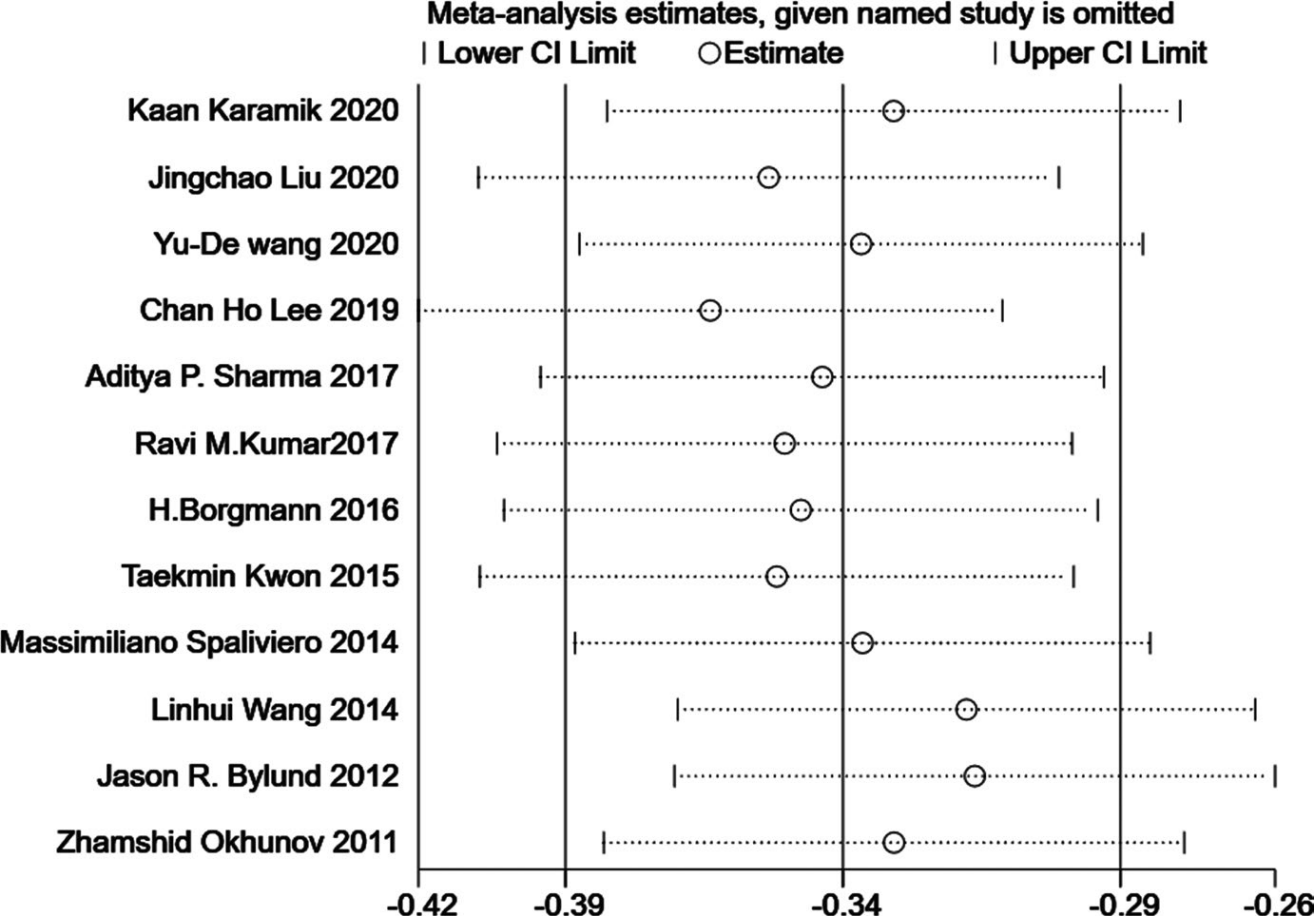
Sensitivity analysis of correlation between centrality index (C‐index) scoring system and warm ischemia time

**FIGURE 11:**
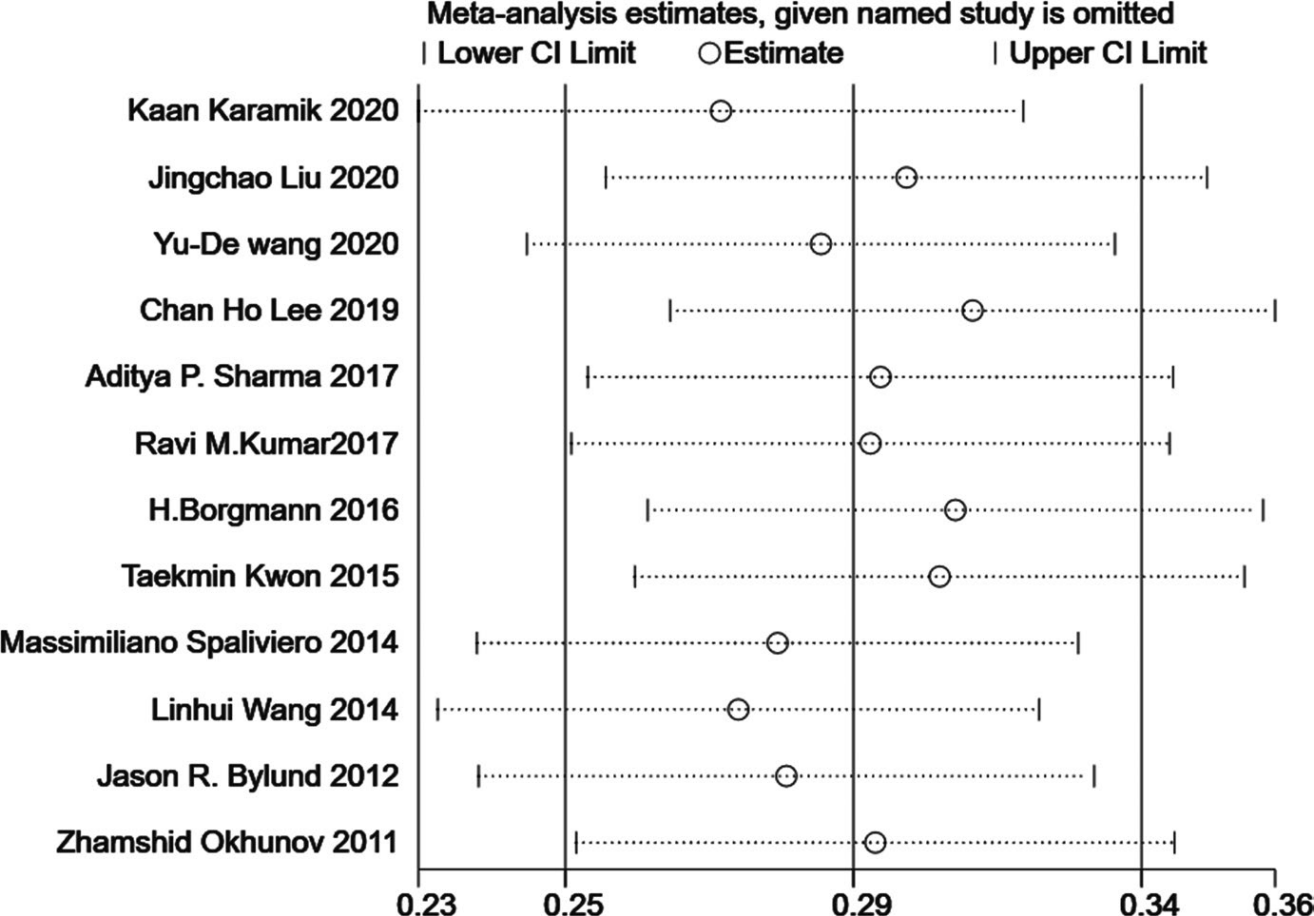
Sensitivity analysis of correlation between the Preoperative Aspects and Dimensions Used for an Anatomical (PADUA) scoring system and warm ischemia time

**FIGURE 12:**
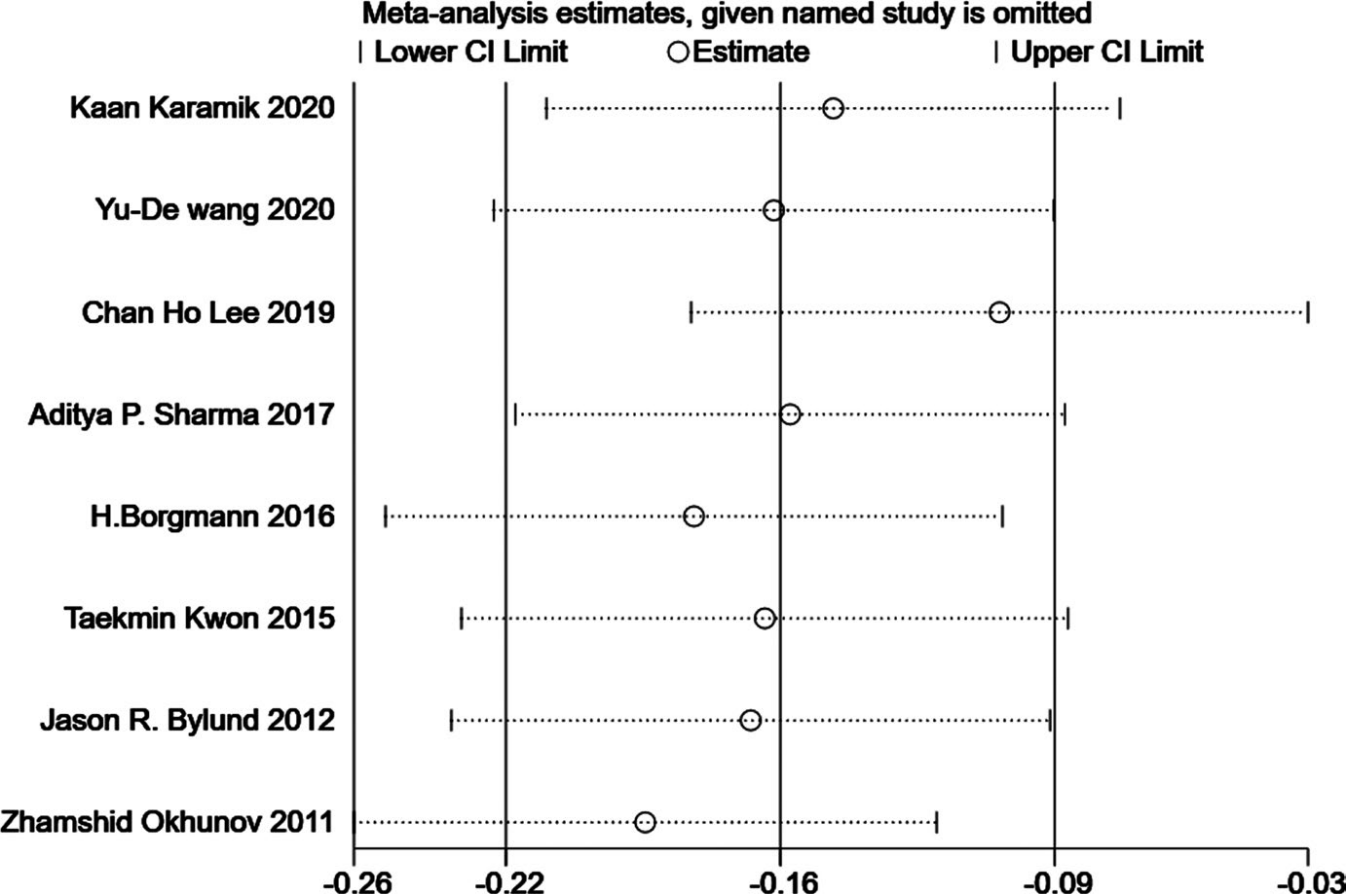
Sensitivity analysis of correlation between centrality index (C‐index) scoring system and estimated blood loss

**FIGURE 13:**
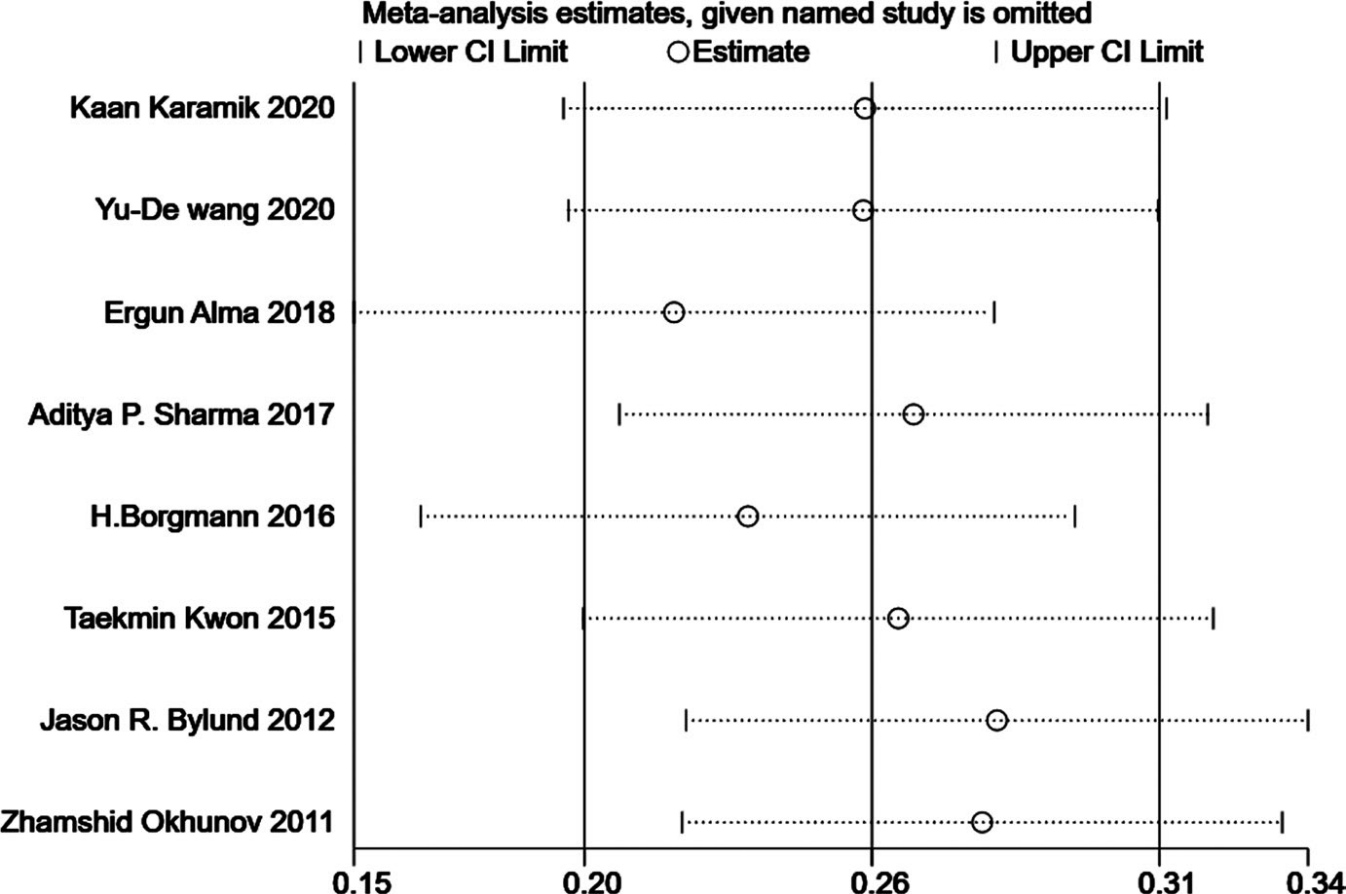
Sensitivity analysis of correlation between the radius, exophytic/endophytic, nearness, anterior/posterior, location nephrometry score (R.E.N.A.L.) scoring system and operation time

**FIGURE 14:**
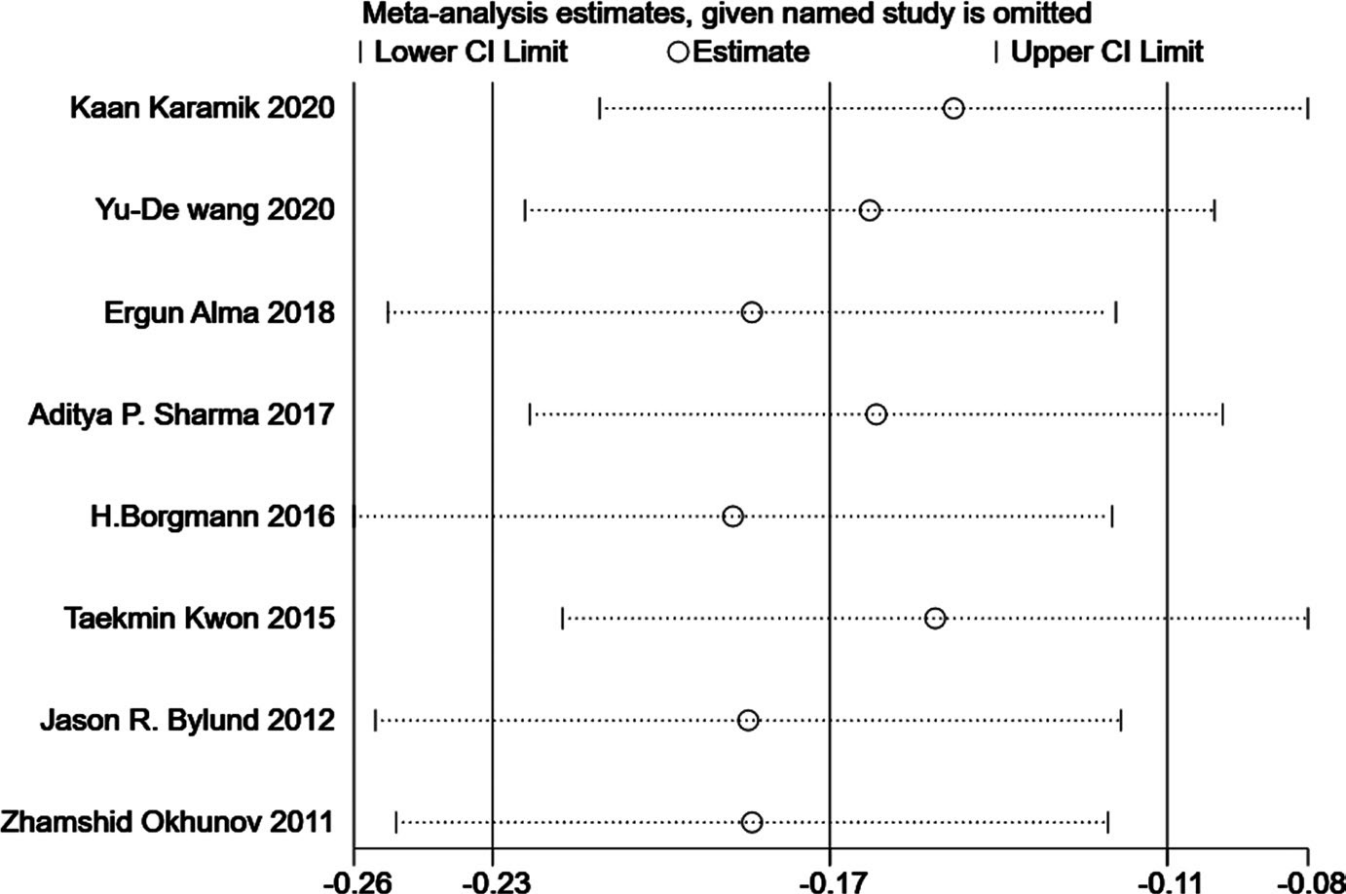
Sensitivity analysis of correlation between centrality index (C‐index) scoring system and operation time

**FIGURE 15:**
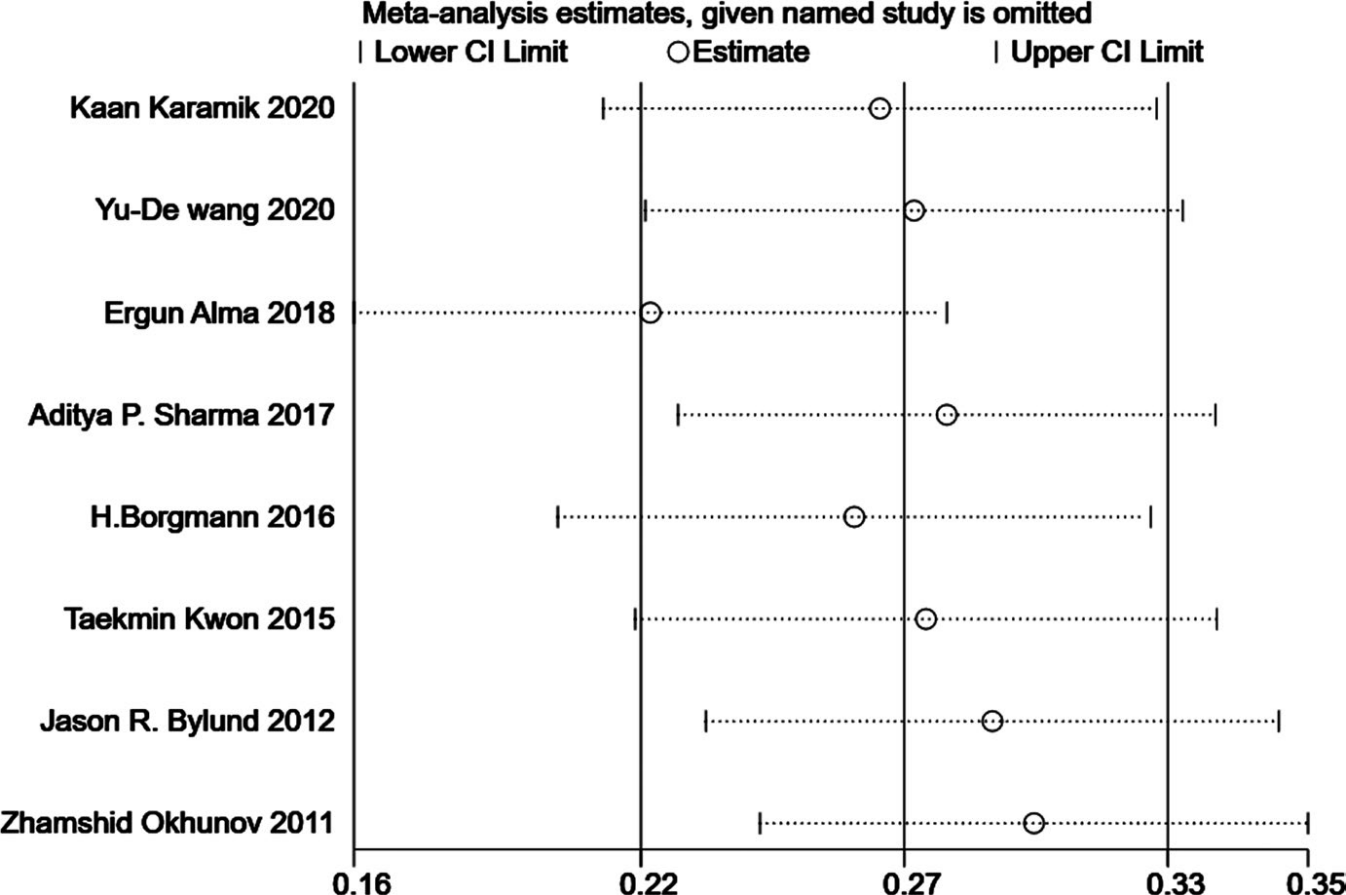
Sensitivity analysis of correlation between the Preoperative Aspects and Dimensions Used for an Anatomical (PADUA) scoring system and operation time

**FIGURE 16:**
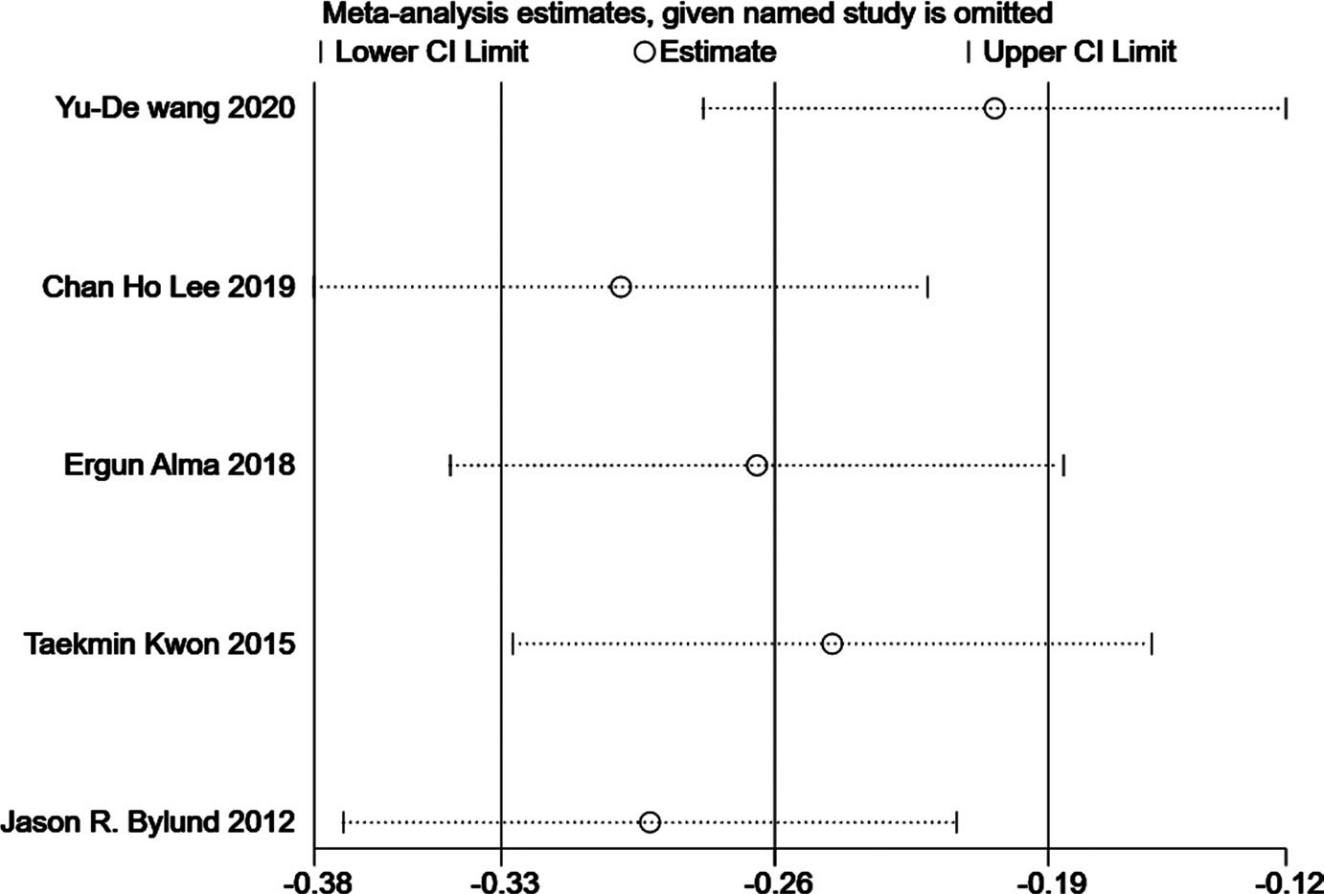
Sensitivity analysis of correlation between centrality index (C‐index) scoring system and absolute change in estimated glomerular filtration rate

**FIGURE 17:**
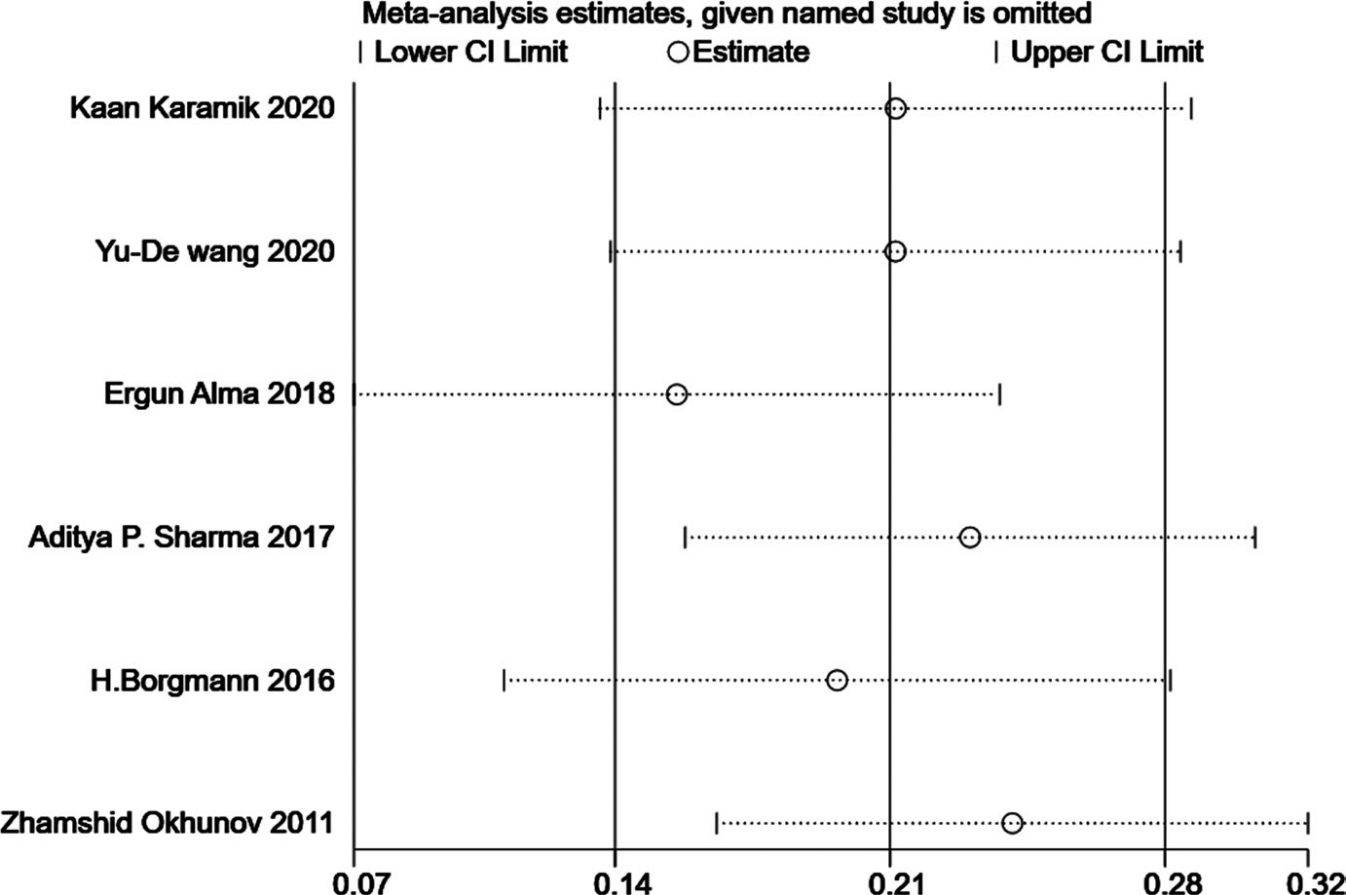
Sensitivity analysis of correlation between the radius, exophytic/endophytic, nearness, anterior/posterior, location nephrometry score (R.E.N.A.L.) scoring system and length of stay

**FIGURE 18:**
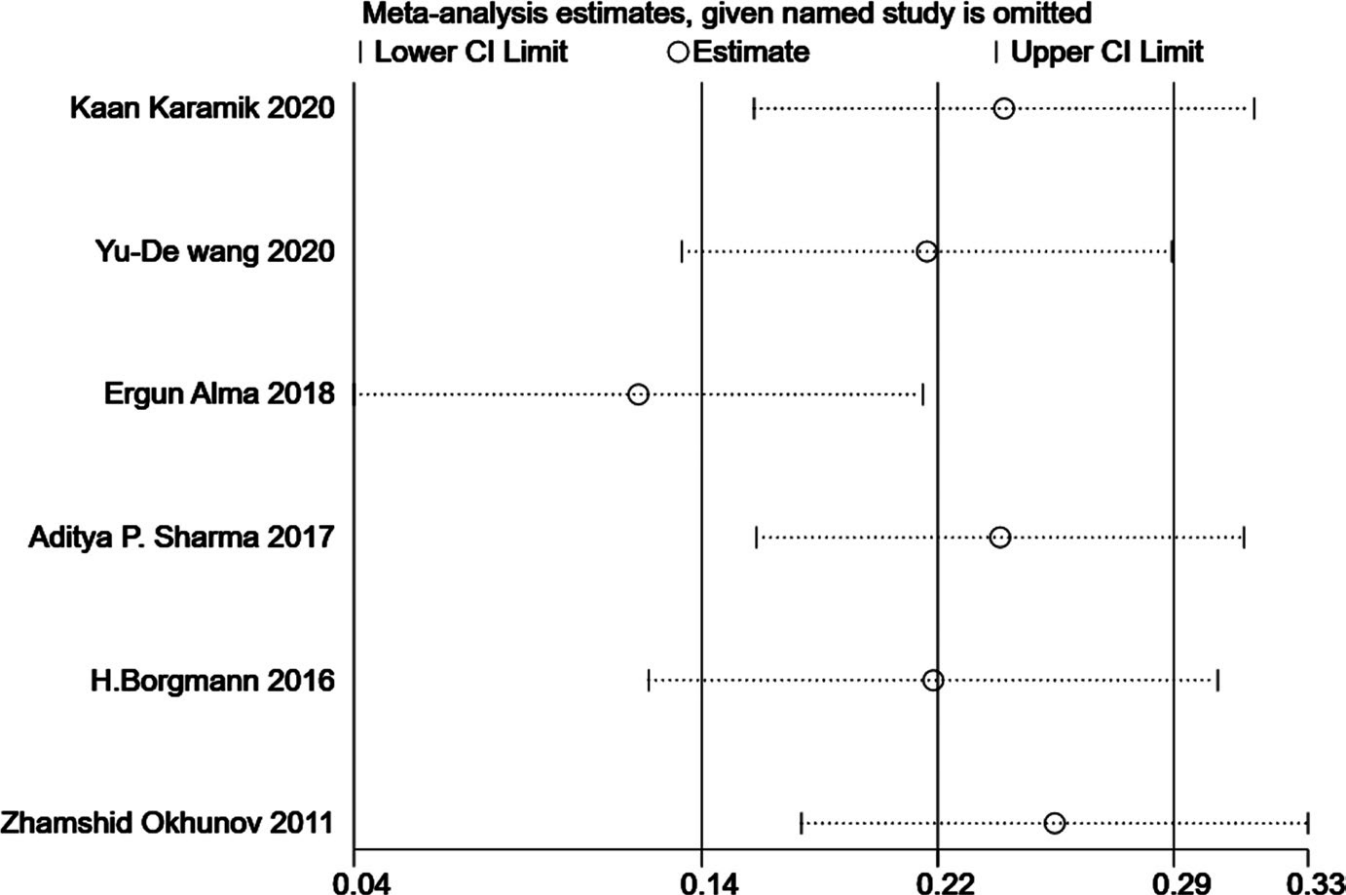
Sensitivity analysis of correlation between the Preoperative Aspects and Dimensions Used for an Anatomical (PADUA) scoring system and length of stay

Since significant heterogeneity was found in our results, sensitivity analysis was performed in Stata to confirm the source of high heterogeneity (Figures 9, 10, 11, 12, 13, 14, 15, 16, 17, 18). For the RENAL (*I* ^2^ = 59.1%), PADUA (*I* ^2^ = 61.7%), and C‐index (*I* ^2^ = 70.1%) scores in WIT, when Lee et al.^24^, Karamik et al.^21^ and Lee et al.^24^ were removed respectively, the results changed (from *r* = 0.29 [0.22, 0.37], *p *< 0.05 to *r* = 0.32 [0.25, 0.39], *p *< 0.05; from *r* = 0.29 [0.21, 0.37], *p *< 0.05 to *r* = 0.27 [0.20, 0.35], *p *< 0.05; from *r* = −0.345 [−0.43, −0.26], *p *< 0.05 to *r* = −0.37 [−0.45, −0.29], *p *< 0.05). For the C‐index (*I* ^2^ = 60.4%) score in EBL, when Lee et al.^24^ was removed, the results changed (from *r* = −0.14 [−0.24, −0.04], *p *< 0.05 to *r* = −0.10 [−0.18, −0.02], *p *< 0.05). For the RENAL (*I* ^2^ = 61.5%), PADUA (*I* ^2^ = 71.1%) scores in OT, when Alma et al.^17^ was removed, the results changed (from *r* = 0.23 [0.13, 0.33], *p *< 0.05 to *r* = 0.20 [0.10, 0.29], *p *< 0.05; from *r* = 0.23 [0.12, 0.35], *p *< 0.05 to *r* = 0.19 [0.09, 0.29], *p *< 0.05). With regard to the C‐index (*I* ^2^ = 52.4%) score in OT, the final result remained similar after the removal of the study by Kwon et al.^23^ For the C‐index (*I* ^2^ = 87.3%) score in ACE, when Wang et al.^32^ was removed, the results changed (from *r *= −0.29 [−0.48, −0.10], *p *< 0.05 to *r* = −0.20 [−0.29, −0.10], *p *< 0.05). For the RENAL (*I* ^2^ = 60.1%), PADUA (*I* ^2^ = 74.0%) scores in LOS, when Alma et al.^17^ was removed, the results changed (from *r* = 0.17 [0.04, 0.30], *p *< 0.05 to *r* = 0.13 [0.00, 0.25], *p *< 0.05; from *r* = 0.16 [−0.00, 0.31], *p *= 0.053 to *r* = 0.10 [−0.03, 0.22], *p *= 0.133). Although the existence of heterogeneity, no obvious impact was observed on the final results except for C‐index score in ACE which we will discuss later.

### 3.4 Publication bias

Egger's test was applied to quantify potential publication bias. No publication bias was found in most analyses (*p* > 0.05) except for the correlation coefficient between PADUA score and ACE (*p* = 0.04).

## 4 DISCUSSION

At present, it is the first systematic review and meta‐analysis that parallelly compares R.E.N.A.L, PADUA, and C‐index scoring systems in predicting the outcomes after PN. Current studies have shown conflicting reports. Some studies demonstrated the correlation of the three scoring systems with perioperative parameters and postoperative renal functional change while others found weak or even no correlation between each system and outcomes after PN.^18^, ^19^, ^28^, ^29^, ^30^ Therefore, in order to provide an evidence‐based reference for clinical judgement, the present study integrated all the literature including the three systems to demonstrate the correlation and which scoring system performed better. The results of our meta‐analysis illustrated that all the three scores had a statistically significant correlation with perioperative outcomes and postoperative renal functional change. The C‐index score was the strongest predictor of WIT and ACE while EBL, OT, and LOS were weakly correlated with the three scores.

As a surrogate of tumor complexity, WIT gets most of our attention among the intraoperative outcomes.^33^, ^34^ In according with others,^35^, ^36^, ^37^ our results indicate that all the three scores were significantly correlated with WIT, but what's special is that the C‐index score relatively outperforms the R.E.N.A.L. score and PADUA score. This interesting finding is most striking in Wang et al.^31^, which found that the correlation was much stronger than others and C‐index score showed the strongest correlation in the overall analysis. They made a comparison with Bylund et al.^19^ and ascribes the difference to the mixed PN surgical approach. In the study of Borgmann et al.^18^, robot‐assisted PN had just recently became a standard approach in their centers. Therefore, only tumors with low complexity were selected for operation, which may lead to a better performance in RPN subgroup. However, recent reports demonstrating that the minimally invasive surgery may increase WIT denied the supposition.^38^, ^39^ Thus, the present study tends to attribute the conclusion to the different Intraclass correlation coefficient (ICC) of the three NSs, which measures inter‐rater agreement reliability. Okhunov et al.^28^ and Hew et al.^40^ found the C‐index score showing a lower ICC than the other two. However, only a small group of reviewers at different stages of professional development assessed the scores in these studies. Spaliviero et al.^30^ published a study that all the scores were evaluated by readers with different experience level. In their study, the highest ICC was found in C‐index score, showing the highest reliability of inter‐rater agreement and seeming to be least subjective. Besides, some components of R.E.N.A.L. score and PADUA score were found high discordance among readers.^28^, ^40^, ^41^ This finding illustrated that further refinement was needed to ensure the reproducibility due to some clinically insignificant components of NSs. Interestingly, most of the studies presenting components in the three scores mentioned tumor size and the involvement of sinus and collecting system, suggesting that the two parameters may contribute to surgical complexity.^19^, ^20^, ^24^, ^25^, ^32^ From what has been discussed above, since Simmons et al.^9^ described the C‐index to quantify the proximity of kidney tumors to the renal central sinus, tumor size and C‐index are recommended to be used for the prediction of WIT and the experience of the reviewers should be considered. Certainly, we are looking forward to more studies conducted to support our analysis. In sensitivity analysis, the high heterogeneity may derive from Lee et al.^24^ We found that this was the only study in which intravenous mannitol administration was used during the renal artery clamping.

Renal function was considered to be another important parameter after PN. Among all the three scores, the C‐index score had the strongest correlation with ACE significantly (*r *= −0.29 [−0.48, −0.10]), with a high heterogeneity (*I* ^2^ = 87.3%). Wang et al.^32^ was confirmed to be the source of heterogeneity and we attached more importance to their study. When their study was removed, the strength of the correlation weakened (from *r *= −0.29 [−0.48, −0.10] to *r* = −0.20 [−0.29, −0.10]). In the study of Wang et al.^32^, 99mTc‐MAG3 was applied to quantify split renal function measured by effective renal plasma flow (ERPF) preoperatively and 1 year after PN, which was deemed to be superior to other indicators of renal function such as 99mTc diethylene triamine penta‐acetic acid (DTPA) and reflected the true changes in renal function after PN.^42^ This difference may explain why the correlation between absolute change in eGFR (ACE) and scores in their study is relatively stronger than others. Then, subgroup analysis was conducted by the two different methodologies of renal function evaluation (ACE evaluated by MDRD or ACE evaluated by radionuclide GFR). Compared with ACE evaluated by radionuclide GFR, the correlation between ACE evaluated by MDRD and C‐index score was much weaker. This difference demonstrated that radionuclide scan was closer to clinical practice and radionuclide GFR outbalanced to MDRD for evaluation of renal function.^43^ Actually, we found that all our included studies were committed to better assessing the true impact of PN on renal function. Owing to compensatory renal hypertrophy in a two‐kidney model and serum creatinine (SCr) levels affected by some physiologic processes (e.g., the rate of creatinine biosynthesis), inconsistencies and inaccuracies have been reported when quantifying eGFR based on SCr.^44^, ^45^ We also noticed that some studies reflected long‐term renal functional results while some had a short follow‐up period. Renal function was evaluated 1 year after surgery by Wang et al.^32^ and 99mTc‐DTPA scans were conducted at 6, 18, and 30 months in the study of Kwon et al.^23^, postoperatively. Besides, in Lee et al.^24^ and Alma et al.^17^ studies, the follow‐up periods were 12 months and 3 or 4 months, respectively. However, in the study of Bylund et al.^19^, postoperative eGFR was calculated during the period from 1 to 6 months after surgery whenever available. Therefore, the different follow‐up periods of studies could be another source of heterogeneity. Due to no unified standards, this difference may not have significant influence on our result.

The correlations between scores and EBL, OT, and LOS were statistically significant, but the correlation coefficients were all relatively weak compared to WIT and ACE. Investigators have published contradictory results on this issue. Corradi et al.^46^ reporting data from 283 patients who underwent RPN, found a significant correlation between the three scores and EBL and LOS. In the study by Kwon et al.^23^, all the three scores were useful for predicting EBL, and at least one could correlate with OT and LOS. However, other investigators demonstrated that none of the scores was able to assess these perioperative outcomes.^19^, ^20^, ^29^ Plausible reasons for the different result and poor correlation of the three NSs with these outcomes were as follows. First of all, these three NSs were primarily devised for quantitating the salient anatomy of renal masses and expected difficulties to be experienced during PN.^7^, ^8^ Nevertheless, the difficulties in clinical practice were determined by a variety of factors such as the different levels of operative experience among surgeons, the diverse individual characteristics of renal hilum anatomy and renal vascular anomalies encountered during PN.^47^ Furthermore, due to the mixed PN surgical approach in most studies (OPN or LPN or RPN; transabdominal or retroperitoneal), types of operation may result in the controversy.^48^, ^49^ In addition, as was shown in the literature investigating the role of obesity in RPN,^50^ characteristics depending on the patients may also lead to higher EBL, longer OT or LOS. The extent of caution in different centers may also be a potential influence on LOS.

A previous meta‐analysis describing all currently available NSs up to April 2019 showed that the RENAL and PADUA scores were easy to calculate and had a good correlation with most outcomes while other mathematical assessment–based NSs were limited by their complexity and lack of evidence supporting their predictive value.^51^ However, the limitation of their study was that they did not compare renal scoring systems parallelly. On the contrary, the advantage of our meta‐analysis was that all the included studies conducted the evaluation of three NSs in the same group of patients, observers and surgeons, which made the conclusion more convincing. In addition, the C‐index score was calculated in all our included studies, which provided evidence for the predictive value of scores based on mathematical models.

It is undeniable that this meta‐analysis has several limitations. First, all our included studies were retrospective studies with nature of biases. Secondly, it was not possible to quantify the influence of surgical strategy on parameters, so the results were only credible for the mixed techniques (OPN or LPN or RPN; transabdominal or retroperitoneal) instead of the specific one. Thirdly, although high heterogeneity existed in our study, we could not find the source of the high heterogeneity of some parameters. Lastly, the scale of studies mentioning the two methodologies of renal function evaluation was relatively small, which was the biggest limitation of our meta‐analysis. Large‐scale and well‐designed studies are warranted for a valid conclusion.

## 5 CONCLUSION

To our knowledge, this is the first study that parallelly compares these three scoring systems in predicting outcomes after PN. Overall, three scoring systems were significantly correlated with WIT, EBL, OT, and ACE. Moreover, the C‐index scoring system outperformed R.E.N.A.L. and PADUA scoring systems in WIT and ACE. Due to the existence of publication bias and high heterogeneity, further high‐quality studies should be conducted to validate the conclusion externally.

## CONFLICT OF INTEREST

The authors have no conflicts of interest to declare.

## ETHICS STATEMENT

This meta‐analysis was conducted ethically in accordance with the World Medical Association Declaration of Helsinki.

## Supporting information

Supporting Information

Supporting Information

## Data Availability

The authors confirm that the data supporting the findings of this study are available within the article and its supplementary materials. All the data were generated from previous studies ranging from Refs [17, 18, 19, 20, 21, 22, 23, 24, 25, 26, 27, 28, 29, 30, 31, 32].
