# Supplementary material for: Parallel comparison of R.E.N.A.L., PADUA, and C‐index scoring systems in predicting outcomes after partial nephrectomy: A systematic review and meta‐analysis
Source: Cancer Med. 2021 Jul 14;10(15):5062–77. doi: 10.1002/cam4.4047 (PMC8335816; doi:10.1002/cam4.4047)
Supplement: Supplementary file 1 — Supporting Information [file CAM4-10-5062-s001.pdf]

Newcastle-Ottawa quality assessment scale

Study	Selection				Comparability	Exposure/Outcome			Total scores
	1	2	3	4	1	1	2	3	
Kaan Karamik (2020)	*	*	*	*	**	*	*	*	9*
Jingchao Liu (2020)	*	*	*	*	*	*	-	*	8*
Yu-De wang (2020)	*	*	*	*	**	*	*	*	9*
Chan Ho Lee(2019)	*	*	*	*	*	*	-	*	7*
Ergun Alma (2018)	*	*	*	*	*	*	-	*	7*
Aditya P. Sharma (2017)	*	*	*	*	**	*	-	*	8*
Ravi M.Kumar (2017)	*	*	*	*	**	*	-	*	8*
H.Borgmann (2016)	*	*	*	*	**	*	-	*	8*
Taekmin Kwon (2015)	*	*	*	*	**	*	*	*	9*
Massimiliano Spaliviero (2014)	*	*	*	*	**	*	-	*	8*
Linhui Wang (2014)	*	*	*	*	*	*	-	*	7*
Jason R. Bylund (2012)	*	*	*	*	**	*	-	*	8*
Zhamshid Okhunov (2011)	*	*	*	*	*	*	-	*	7*
