# Supplementary material for: Parallel comparison of R.E.N.A.L., PADUA, and C‐index scoring systems in predicting outcomes after partial nephrectomy: A systematic review and meta‐analysis
Source: Cancer Med. 2021 Jul 14;10(15):5062–77. doi: 10.1002/cam4.4047 (PMC8335816; doi:10.1002/cam4.4047)
Supplement: Supplementary file 2 — Supporting Information [file CAM4-10-5062-s002.pdf]

Pubmed:

((nephrometry) OR (The radius, exophytic/endophytic, nearness, anterior/posterior, location score) OR (R.E.N.A.L.) AND (the Preoperative Aspects and Dimensions Used for an Anatomical score) OR (PADUA) AND (centrality index) OR (C-index)) AND ((Partial Nephrectomy) OR (nephron sparing surgery))

WOS:

((The radius, exophytic/endophytic, nearness, anterior/posterior, location score) OR (R.E.N.A.L.) AND (the Preoperative Aspects and Dimensions Used for an Anatomical score) OR (PADUA) AND (centrality index) OR (C-index)) AND (nephrometry) AND ((Partial Nephrectomy) OR (nephron sparing surgery))

Ovid:

Step	Search term	number
1	nephrometry.af.	1730
2	(RENAL or the radius, exophytic, nearness, anterior, location score).af.	1872315
3	(PADUA or the Preoperative Aspects and Dimensions Used for an Anatomical).af.	58982
4	(C-index or centrality index).af.	12105
5	(Partial Nephrectomy or nephron sparing surgery).af.	21811
6	1 and 2 and 3 and 4 and 5	131

Embase:

History	Search term	number
#1	nephrometry	1550
#2	renal OR (the AND radius, AND exophytic, AND nearness, AND anterior AND location AND score)	985337
#3	padua OR (the AND preoperative AND aspects AND dimensions AND used AND for AND an AND anatomical AND score)	42094
#4	'c index' OR (centrality AND index)	8103
#5	partial AND nephrectomy OR (nephron AND sparing AND surgery)	20523
#6	#1 AND #2 AND #3 AND #4 AND #5	56
